# Sexual Dysfunction and Self Esteem Among Tunisian Women

**DOI:** 10.1192/j.eurpsy.2025.424

**Published:** 2025-08-26

**Authors:** N. H. Benhamed, A. Hakiri, Y. Yaich, B. Amamou

**Affiliations:** 1 psychiatry department, EPS Fatouma Bourguiba, Monastir; 2 Psychiary B, Razi Hospital, Manouba, Tunisia

## Abstract

**Introduction:**

Sexual dysfunction is a multifaced issue that significantly affects women’s physical and psychological health, contributing to broader emotional challenges such as dissatisfaction and inadequacy. In Tunisia, cultural and social factors, including gender roles and societal expectations, further shape women’s perceptions and experiences of their sexual well-being. Self-esteem, a core component of psychological health, plays a crucial role in how women view and experience their sexuality, with lower self-esteem often intensifying sexual dysfunction.

**Objectives:**

This study aims to explore the relationship between sexual dysfunction and self-esteem among Tunisian women within this cultural context.

**Methods:**

a cross-sectional study was conducted online using a Google Forms questionnaire between July and August 2024.The inclusion criteria were sexually active women aged 18 years or older who provided informed consent to participate. Participants completed a self-administered questionnaire that included sociodemographic information, personal medical history, lifestyle habits, and psychometric assessments.

The Female Sexual Function Index (FSFI) was used to evaluate sexual function and self-esteem was assessed using the Rosenberg Self-Esteem Scale (RSE).

**Results:**

A total of 180 women participated in the study. The average age of the sample was 32.79, with ages ranging from 21 to 60 years. In our study, 97.78% of the women were from urban areas 94.44% had a university degree 80% were employed and 62.78% were married. Regarding medical history, 21.11% reported organic issues, while 27.22% had a psychiatric history.

Lifestyle habits indicated that 18.9% of women smoked, and 21.1% consumed alcohol, while only 1.1% reported using psychoactive substances. . The majority, 93.89%, had a single partner, and 93.89% identified as heterosexual.

The evaluation of sexual function using the Female Sexual Function Index (FSFI) showed an average score of 23.37 ± 9.64, with 43.33% of participants experiencing sexual dysfunction. Specifically, 75.6% had issues with sexual desire, 83.3% reported pain during intercourse, and 71.7% experienced problems with sexual arousal.

The average self-esteem score, was 32.25 ± 5.75. A significant correlation was found between sexual dysfunction and self-esteem (p < 10^-3). Among the women with very low self-esteem, 80% experienced sexual dysfunction, while only 20% of those with very high self-esteem reported dysfunction.

**Conclusions:**

Addressing both sexual health and self-esteem is essential for improving the emotional and psychological well-being of women in Tunisia. These findings underscore the importance of comprehensive sexual health interventions that promote positive self-esteem, ultimately enhancing the overall quality of life and fostering psychological resilience among Tunisian women.

**Disclosure of Interest:**

None Declared

